# Solidarity or Segregation? ChatGPT Health and US Health Care Disparities

**DOI:** 10.2196/94972

**Published:** 2026-06-22

**Authors:** Andrew J Barnhart, Giuseppe Comerci, Barbara Prainsack, Matthias Braun

**Affiliations:** 1 Department of Social Ethics, University of Bonn, Am Hofgarten 8, Bonn, North Rhine-Westphalia, 53113, Germany; 2 Department of Political Science, University of Vienna, Vienna, Austria

**Keywords:** large language models, digital health policy, solidarity, artificial intelligence, AI, health care inequity

## Abstract

ChatGPT Health, an artificial intelligence feature launched by OpenAI in January 2026, integrates personal medical records and consumer health data into a large language model–based chatbot. It positions itself as a digital health tool to help users navigate a fragmented US health care system marked by inaccessible insurance, medical deserts, and workforce shortages. We argue that, rather than closing these gaps, ChatGPT Health risks widening what we call the solidarity gap in health care. By appearing to address unmet needs, it may obscure and entrench the structural conditions that produce health care disparities. We identify specific risks to patient safety, such as the legitimization of dangerous self-rationing and self-medication behaviors, inaccurate triage of clinical emergencies, erosion of effective health communication, and the reinforcement of confirmation and anchoring bias. These risks are particularly acute for individuals facing structural barriers to care. To prevent ChatGPT Health and similar large language model–based health tools from deepening inequities, we propose solidarity-based policy interventions such as upstream reforms that rebuild the institutional foundations of access and health equity and downstream safeguards that embed artificial intelligence tools within accountable, equitable health care infrastructures as complements to rather than substitutes for human care.

## Introduction

Overburdened health care services and multiple therapeutic gaps leave many health needs unmet. Many of those with unmet needs are now turning to artificial intelligence (AI) to fill in these therapeutic gaps themselves, which in turn may unburden health care services [1]. OpenAI, a US-based company developing advanced commercial and consumer AI, published a report in January 2026 that illustrated how people used ChatGPT for health care–related purposes [2]. According to the report, more than 40 million users worldwide consult ChatGPT daily with health-related questions. In the United States alone, nearly 2 million queries per week concern health insurance, whereas in rural communities, approximately 600,000 weekly messages relate to health care. Seven in 10 health care conversations occur outside clinical hours, showing a persistent demand for information and support [2]. Another study of over 500,000 deidentified Microsoft Copilot conversations in January 2026 found that nearly 1 in 5 conversations involved personal symptom assessments or discussions regarding conditions [3]. These conversations rose in frequency in the evening and nighttime hours, times when in-person health care services are less available [3].

For ChatGPT or other large language model (LLM) users who have access to health care, services and data are often fragmented: a laboratory test PDF in one portal, visitation notes in another, symptoms and health data tracked through an app, and no clear way to connect it all for a bigger picture. ChatGPT Health, launched in January 2026, is a new feature of OpenAI’s chatbot that attempts to solve the first 2 challenges identified in the report: supporting users in navigating health care systems and helping mitigate access gaps [4]. OpenAI frames their Health feature as a guide through the uncertainty of the US health care system [4]. However, embedding this capability within ChatGPT is a double-edged sword. While it may be of help for some, in other contexts, the Health feature may fall short of closing the intended care gaps and could inadvertently amplify hazardous attitudes and behaviors among users.

When viewed through a governance lens, ChatGPT Health reframes what support looks like. The feature does not change the affordability of health insurance, availability of appointments, or in-person clinical workforce capacity. However, it does change how a person interprets and engages with their health data and health-related information more broadly. ChatGPT Health is designed to unite personal medical information (eg, laboratory results, medical histories, PDFs, and notes) with consumer health data from apps such as Apple Health, Function Health, and MyFitnessPal [4]. By aggregating these sources, ChatGPT Health can generate responses grounded in a user’s own data, promising an overall wellness snapshot, simplifying test results, or proposing a personalized training plan. It presents itself as a tool that reduces administrative complexity for the individual. However, at the same time, it raises questions about the responsibility of health data interpretation, whether this role will create conflicts with medical expertise and clinical institutions, and whether it shifts responsibility toward the individual [5,6].

Centralizing sensitive personal data within ChatGPT Health comes with privacy and security risks. To mitigate this, ChatGPT Health operates as a distinct module with dedicated memory, keeping medical conversations separate from general ChatGPT chats. Sensitive information does not flow into nonhealth interactions, although ChatGPT Health may use relevant context from prior conversations to improve coherence [4]. For instance, when explaining recurrent high blood pressure, it could consider an earlier discussion about job loss as a potential stressor. Given the sensitivity of medical data, ChatGPT Health adds privacy and security measures beyond those of standard ChatGPT, including purpose-built encryption and optional multifactor authentication [4]. Users retain control and can disconnect apps at any time or delete ChatGPT Health’s memory.

At the time of writing, OpenAI has begun providing access to ChatGPT Health to a limited group of testers to refine the experience [4]. Partly due to strict data protection rules in Europe, the service is currently offered only outside the European Economic Area, Switzerland, and the United Kingdom. Correspondingly, despite OpenAI’s stated commitment to making ChatGPT Health available to all ChatGPT users, the system appears to be optimized primarily for users in the United States. To aggregate medical data, OpenAI relies on b.well, a US-based platform for health data management [4]. As a result, the integration of medical data—intended as a core component of the ChatGPT Health feature—with consumer-facing applications currently seems to be available only in the United States.

Importantly, ChatGPT Health is not the only LLM-based tool entering the health care space. Comparable systems—such as Claude for Healthcare (Anthropic), Perplexity Health (Perplexity), and Copilot Health (Microsoft)—have recently been announced with similar functionalities [5-7]. While this paper focuses on ChatGPT Health, the dynamics and challenges discussed are likely to extend to LLM-based health tools more broadly.

ChatGPT Health is expected to have an immediate and major impact on the US health care landscape. Analyzing its deployment in this context can help anticipate broader implications and potential challenges that may emerge as similar systems are introduced in other countries, making the United States a particularly instructive case study.

Our core argument is that ChatGPT Health, along with similar LLM health modes, risks widening the solidarity gaps in US health care. While they may appear to address unmet needs, such chatbots can obscure and entrench the structural disparities that led to those unmet needs in the first place. These chatbot health interventions risk dangerous self-rationing and self-medication behaviors, inaccurate triage, erosion of effective health communication, and the reinforcement of confirmation bias. We propose that these gaps cannot be closed with digital tools. Instead, reducing these gaps requires solidarity-based policy interventions at 2 levels: upstream reforms that strengthen the institutional foundations of access and affordability and downstream safeguards governing the use of AI tools such as ChatGPT Health within the health care system.

## Inaccessible Health Insurance and Health Care in the United States

OpenAI draws attention to 2 systemic issues in US health care [4]. First, health care is hard to navigate: unclear, convoluted information makes decisions (such as choosing an insurance plan) confusing and frustrating. ChatGPT already receives nearly 2 million health insurance messages each week [2]. Access and affordability are worsening as federal subsidies lapse. At the end of 2025, enhanced premium tax credits and cost-sharing reductions enacted by the American Rescue Plan and extended by the Inflation Reduction Act expired, threatening the affordability of Health Insurance Marketplace coverage for people ineligible for Medicaid and without affordable employer plans. Congress is debating whether to reinstate, extend, or make these subsidies permanent [7], although news outlets report that talks have diminished, with no agreed-upon action [8]. Without the subsidies, premiums are projected to more than double and, in some cases, quintuple, pricing out millions of people [7]. The Urban Institute estimates 4.8 million additional uninsured people in 2026 if subsidies are not extended, leaving many Americans more vulnerable in their health and care [7].

The second issue underscored by OpenAI is so-called medical deserts [2]. These are areas where residents lack adequate access to essential services such as physicians, pharmacies, hospitals, and specialists [9]. Contributing factors include work conditions, lifestyle, health care workforce migration, and sociodemographics [9]. The crisis is acute. Since 2010, more than 150 rural hospitals have closed or ended inpatient care, and over 400 face potential closure due to financial instability [10]. Less profitable services such as emergency care, obstetrics, and behavioral health are often cut first [10]. Uneven workforce distribution is also linked to worse outcomes. Many factors contribute to the uneven distribution of health care personnel across the territory: working conditions, workload, salary, and career prospects are generally better in urban areas, resulting in a lower presence of health care personnel in rural areas [7]. As a consequence, patients farther from facilities have lower survival rates, longer hospital stays, and higher rates of missed follow-ups [9]. When structural deficiencies and disparities such as these exist, it seems only natural that those who need health care would gravitate toward a more readily available alternative. And so, we now turn our attention to chatbots and digital health.

## Limitations and Plausible Risks of ChatGPT Health Interventions

OpenAI presents ChatGPT Health as a practical tool to help address these shortcomings [2]. With only a smartphone and an internet connection, it is widely accessible and could temporarily mitigate some structural gaps. At the same time, the “cure” that ChatGPT Health promises also poses considerable risks. In what follows, we distinguish between (1) documented structural inequities in health care, (2) known and empirically established limitations of LLMs, and (3) anticipated risks that follow plausibly from points 1 and 2 but have not yet been fully empirically established.

The first concern relates to people with poor or no access to health care services. We anticipate that, here, ChatGPT Health could create a perverse effect. While appearing to mitigate the problem, it may make matters worse by giving false reassurance through persuasive, humanlike, empathetic responses that a chatbot is a suitable stand-in for advice from an experienced and adequately trained professional. For instance, it is well documented that adults with obesity have struggled to access glucagonlike peptide-1 receptor agonists due to limited insurance coverage, leading some to ration use to cut costs [11]. In a plausible scenario enabled by ChatGPT Health, patients who cannot afford medications promptly or who lack access to primary care or pharmacies might turn to ChatGPT Health for advice on questions such as “What happens if I miss a daily dose?” or “What symptoms could occur if I delay my medication by eight hours?” If the answers suggest low immediate risk, patients may feel reassured and choose to skip or delay doses. In this way, ChatGPT Health could provide reliable information that inadvertently legitimizes self-rationing. This practice is risky and potentially fatal for conditions that require continuous treatment, such as diabetes [11].

Another likely consequence of overreliance on ChatGPT Health is self-medication, treating symptoms without clinician supervision. A US survey found that nonprescription antibiotic use is common: 43.6% (246/564) of respondents reported prior use without a prescription [12]. Reported drivers included lack of transportation (echoing medical deserts), lack of insurance, and the cost of physician visits [12]. These documented drivers suggest that ChatGPT Health could become an additional pathway to self-medication. Although OpenAI states that ChatGPT Health is not intended to replace clinicians [2], vulnerable users without immediate access to care may use it to justify self-medication. Indirect prompts such as “What should I take for pain from a suspected fracture?” or “Are antibiotics helpful for burning with urination?” could elicit consistent, seemingly actionable guidance.

Self-medication compounds risk because queries about a drug’s suitability often entail self-diagnosis. The likelihood of misdiagnosis is high, and inappropriate medication can cause serious harm. In addition, empirical evidence already indicates concrete triage failures. A study published in *Nature Medicine* found that the ChatGPT Health system’s accuracy followed an inverted U-shaped pattern, meaning that it performed worst at clinical extremes, where errors matter most [13]. Most alarmingly, over half (51.6%) of true emergencies were undertriaged, with the system recommending that patients experiencing conditions such as diabetic ketoacidosis or impending respiratory failure seek care within 24 to 48 hours rather than going immediately to the emergency department [13].

Another risk, especially for people without access to formal care, is the erosion of effective health communication. ChatGPT is a sophisticated language model that generates humanlike text but lacks genuine emotional intelligence and cannot fully grasp the implications of its responses as it lacks contextual knowledge [14]. In a digital-only format, it misses nonverbal cues such as facial expressions and body language, making authentic bidirectional feedback difficult and prone to distortion [14]. More broadly, there is a documented gap between theories of effective health communication and their application in the capabilities of chatbots [15]. Effective health communication relies on social constructs; community dynamics; and identifiable, relatable figures that help individuals envision healthier alternatives, all of which are not fully present in chatbot communications [15]. Poor health communication is particularly risky for individuals in vulnerable psychological states, including those with suicidal ideation, because chatbots cannot truly understand, empathize, or fully provide emotional support [14].

ChatGPT Health may also reinforce confirmation or anchoring bias, particular risks that build from established LLM limitations. Research on LLM-mediated information seeking has established that generative AI’s personalized, conversational style can strengthen existing beliefs, obscure medical consensus, and spread misinformation [13,16]. We anticipate that these dynamics are likely to be amplified within ChatGPT Health. Users often frame questions to fit their preconceptions, and the model tailors answers to those frames rather than nudging toward neutral, evidence-based perspectives. This may normalize behaviors such as self-medication or self-triaging [13,16]. Opaque customization may create echo chambers, and the model’s authoritative tone encourages uncritical acceptance. Compounding the problem, ChatGPT has produced contradictory answers to the same query depending on phrasing, yet both may appear equally convincing to users with limited health literacy [16].

**Figure 1.**
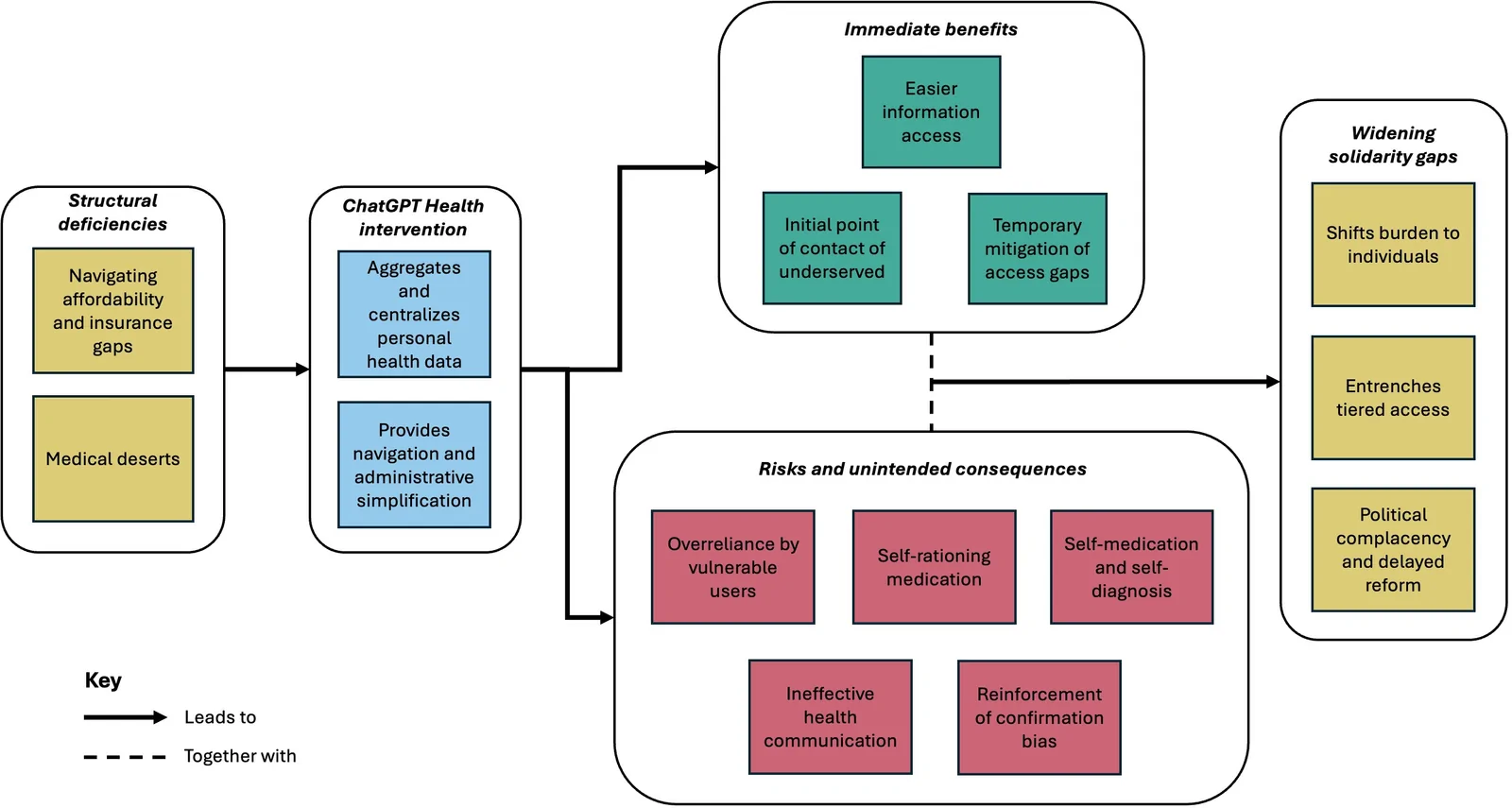
A process flow diagram depicting how ChatGPT interventions can lead to widening solidarity gaps.

While the limitations and risks briefly outlined above are anticipated to be particularly acute for individuals facing structural gaps to care, they are not exclusive to underserved populations. Risks of confirmation and anchoring biases are present regardless of a person’s insurance status or access to a clinician. Inaccurate triage affects any user who consults a chatbot before deciding whether to seek emergency care. However, what distinguishes structurally underserved populations is the clear absence of fundamental safety nets such as trustful relationships with clinicians and a functioning and well-funded health care infrastructure that could otherwise catch and correct errors [17,18]. Without such safety nets, individuals become more vulnerable and, thus, would (at least plausibly) reach for the closest and most easily accessible health resource available—such as ChatGPT Health. In doing so, they can receive immediate benefits such as “health advice,” but they are also taking a risk of greater impacts of harms [19]. Taken together, this leads to a widening of what we call solidarity gaps in health care, as shown in Figure 1.

## Closing, Not Exacerbating, the Solidarity Gap in Health Care

Solidarity in health care is a practice-based commitment to carry costs and share benefits with others, grounded in the recognition of relevant similarities between people—in this case, every person’s vulnerability to disease or injuries [20,21]. Health care systems in Europe and in some other parts of the world [22] have historically been organized around principles of solidarity, whereas the US health care system has largely not. This has produced a situation in which collective support is unevenly realized through governance and access. Tools such as ChatGPT Health risk exacerbating this gap by creating the appearance that unmet needs in health care are being addressed without altering the institutional arrangements that produce unequal access in the first place. Where people must choose between long waiting times, long-distance travel, substantial out-of-pocket payments, or consulting a chatbot, the solidarity gap increases [20]. We define a solidarity gap here as a disparity between the ideal of collective, shared responsibility for health care costs and benefits and the reality of how unevenly collective support is distributed. Furthermore, ChatGPT Health is likely to shift even more burdens onto patients and their families (an anticipated risk that builds on documented patterns of cost shifting in digital health more broadly). It may entrench tiered access where some receive clinician-led care augmented by AI whereas others rely only on a chatbot and foster political complacency that delays investment in clinics, workforce, and coverage. This does not mean that there is no role for ChatGPT and other LLM-based tools in health care. If used and regulated within a well-funded, well-trained, and well-rested workforce within an equitable health care system, such tools can complement human intelligence and experience.

How do we get there? Avoiding a widening of the solidarity gap requires policy that puts solidarity at the center of health system design. Solutions operate both upstream and downstream of tools such as ChatGPT Health. Upstream interventions target the institutional tier of solidarity and are inseparable from justice: they counter structural arrangements that systematically produce inequitable access and outcomes [23]. Addressing medical deserts and insurance gaps requires rebuilding the institutional conditions of accessibility and affordability that make last-resort reliance on chatbots unnecessary. A solidarity-oriented upstream agenda establishes universal, equitable access as a baseline; treats health inequities as products of structural injustice; and expands democratic participation in health system governance so that benefits and burdens are shared fairly. There are proposed democratic participation mechanisms in the context of AI and health policy that could be adapted for the governance of consumer health AI. One such mechanism includes citizens’ juries, a form of deliberative democracy that can obtain informed public judgment on health AI [24] or health policy generally [25].

Moreover, solidarity-based approaches would call for an interconnected distribution of resources that guarantees access to health care also for people living in rural and other underserved areas. For example, consistent rotation of mobile clinics, namely, medical units that provide medical care via vehicles, can be implemented. Services offered by mobile clinics generally focus on primary care and can span screenings, vaccinations, dental checks, and mental health [26]. A further key move could be the introduction of educational requirements in medical programs that include mandatory training in rural areas. In this way, in addition to helping address the shortage of professionals, young students could enrich their training by dealing with unfamiliar contexts [27]. From a more general standpoint, local governments should undertake investments to establish medical infrastructures and facilities and level out discrepancies in the system, guaranteeing democratic and fair access to health care.

Downstream, ChatGPT Health should be embedded in public and clinical infrastructures as a complement to, not a substitute for, human experience, expertise, and care. Evidence indicates that LLMs are most effective in the hands of subject matter experts who can leverage time savings while identifying errors [28]. Therefore, deployment should prioritize augmentation of clinicians and care teams rather than shifting the burden to patients. Ideally, this would directly address the erosion of health communication identified above. Clinicians can read nonverbal cues, apply emotional intelligence, and contextualize responses and, thus, can more readily communicate accurate health information to a patient than a chatbot alone. However, a caveat is that clinicians must not themselves fall prey to the generative pretrained transformer bias risks or cognitive offloading.

Integration should enable warm handoffs to human services, countering the risks of self-rationing and self-medication behaviors described above. However, it is important to note that these service handoffs do not, on their own, address the material reasons for why an individual might initially undertake such courses of action. Safety and equity audits should specifically target triage failures, and unless or until an established threshold of accurate triage is met, the use of ChatGPT Health should be avoided for such purposes entirely. Transparency about model capabilities and limits helps users recognize when chatbot guidance is insufficient, which may mitigate the risks of uncritical acceptance of outputs, and performance evaluations should center on reductions in unmet needs and improved access rather than engagement metrics.

Data governance must likewise be solidarity based. Individual control alone cannot correct power asymmetries between data processors and data subjects or address group-level harms and benefits [26]. Governance should also strengthen collective oversight so that the benefits and costs of data practices are shared fairly and assess data uses by whether they create public value without imposing grave risks [29]. This form of data governance is especially salient for the corresponding LLM companies as they attempt to centralize personal health data while also shifting the interpretative responsibility onto the individual end user. It is salient insofar as it attempts to rebalance such power asymmetries, offers collective data ownership and control, and prioritizes public value. Tools such as PLUTO (Public Value Assessment Tool) serve as practical mechanisms for these value assessments by categorizing data use based on implications for public value while considering power dynamics and sustainability [30]. High–public value, low-risk uses should receive public support; low–public value, high-risk uses should be prohibited and subject to strong enforcement. Because harm can still occur and commercial actors can profit from data use, policy should provide accessible remedies and require that a portion of commercial gains be returned to the public domain [29]. Accessible remedies can take institutional forms such as that of harm mitigation bodies; such bodies can provide support to those who were plausibly harmed by data use and monitor potential harms from big data practices [31]. The return of commercial gains can take the form of data access levies on commercial owners of consumer health AI. The generated revenue could then be redirected to rebuild and maintain traditional health infrastructures and institutions or else put to better use for the people and communities that enabled the success of consumer health AI [29]. In Table 1, we summarize these possible solidarity-based policy interventions and what they target.

**Table 1.** Upstream and downstream solidarity-based policy interventions and what they target and/or prevent.

Upstream or downstream	Solidarity gap addressed	Solidarity-based policy intervention	What it targets or is meant to prevent
Upstream	Institutional access	Rebuild institutional conditions of accessibility and affordability	Prevent chatbot use from becoming a de facto substitute for real coverage, appointments, pharmacies, and in-person capacity
Upstream	Coverage equity	Establish universal, equitable access as a baseline	Prevent tiered access where some receive clinician-led care vs those who rely on chatbots
Upstream	Democratic participation	Expand democratic participation in health system governance	Prevent political complacency and delays in investments in clinics, workforces, and coverage
Downstream	Accountability	Only embed chatbots within equitable systems with accountable infrastructures	Prevent the shifting of responsibility for interpretation and care onto (vulnerable) individuals
Downstream	Measurement	Evaluate performance through reductions in unmet needs and improved access	Prevent incentives that prioritize chatbot use over institutional access
Downstream	Data governance	Data solidarity governance: strengthen collective oversight and assess data uses based on public value vs grave risks	Correct power asymmetries between data processors and data subjects; address group-level harms and benefits

## Conclusions

The appearance of a health-dedicated function in ChatGPT and similar LLMs deserves careful scrutiny. The promise of filling health care gaps risks colliding with health care systems such as those in the United States and potentially widening those gaps. The potential overreliance on ChatGPT Health by individuals experiencing unequal access to health care services calls for solutions rooted in solidarity. However, a solidaristic health system does not emerge from technology alone, but technology can help only if institutions do their part. Policymakers have an obligation to rebuild and strengthen the foundations of access and affordability and embed AI tools only within accountable, equitable infrastructures. If done correctly, ChatGPT Health can complement rather than replace human care and improve navigation without widening gaps. The measure of success cannot be based on LLM engagement. Instead, the measure should be fewer unmet needs, safer care, and easier access to basic health care infrastructure.

What is unique about the concept of a solidarity gap is that it shows how digital health technologies can mask and entrench structural disparities by appearing to meet needs without altering the conditions that produce them. The concept, in a way, forces a shift in the analysis from a tool in isolation to its interaction with the institutional and social contexts in which it is deployed. Moreover, there are clear upstream and downstream policy reforms that can be put in place and that are based on a principle of solidarity. If convenience and the ideal of short-term cost savings replace solidarity, AI, no matter the form, will become a veneer over deepening inequities. Solidarity is the governing principle that will become the bridge to better care.

## Abbreviations

AI: artificial intelligence
LLM: large language model
PLUTO: Public Value Assessment Tool
